# Systemic lupus erythematosus associated with sickle-cell disease: a case report and literature review

**DOI:** 10.1186/1752-1947-6-366

**Published:** 2012-10-26

**Authors:** Mouna Maamar, Zoubida Tazi-Mezalek, Hicham Harmouche, Wafaa Mounfaloti, Mohammed Adnaoui, Mohammed Aouni

**Affiliations:** 1 Department of Internal Medicine, Ibn Sina Hospital, Rabat, Morocco

**Keywords:** Sickle-cell disease, systemic lupus erythematosus

## Abstract

**Introduction:**

The occurrence of systemic lupus erythematosus has been only rarely reported in patients with sickle-cell disease.

**Case presentation:**

We describe the case of a 23-year-old North-African woman with sickle-cell disease and systemic lupus erythematosus, and discuss the pointers to the diagnosis of this combination of conditions and also present a review of literature. The diagnosis of systemic lupus erythematosus was delayed because our patient’s symptoms were initially attributed to sickle-cell disease.

**Conclusions:**

Physicians should be alerted to the possible association of sickle-cell disease and systemic lupus erythematosus so as not to delay correct diagnosis and initiation of appropriate treatment.

## Introduction

Sickle-cell disease (SCD) is a prevalent genetic disorder that includes sickle-cell anemia (the homozygous and most common form of SCD (SS)), sickle-cell hemoglobin C (SC) and sickle-cell β thalassemia (S/β thal) [1]. The protean clinical features of SCD result from chronic variable intravascular hemolysis and microvascular ischemia, leading to damage in multiple organs [2]. The occurrence of connective tissue diseases, in particular systemic lupus erythematosus (SLE), has only been rarely reported in patients with SCD [2]. The incidence of SLE in patients with SCD is not known because most of the published studies are case reports. Due to similar clinical manifestations, diagnosis of SLE in patients with SCD may be difficult and is often delayed. We report the case of a patient who developed symptoms initially attributed to SCD, but on further investigation underlying SLE was revealed.

## Case presentation

A 23-year-old North-African woman with no family history of SCD was admitted to our department of internal medicine with symptoms of anemia, bone pain, arthralgia and fever. Her symptoms had been developing for six weeks with alteration of her general condition and abdominal pain. On physical examination our patient was pale, she had a temperature of 39.5°C, her blood pressure was 130/75mmHg and heart rate was 100 beats/minute. The patient had slight splenomegaly, pain on pressure in the long bones and arthritis in her knees.

Blood test results showed normocytic anemia at 6.6g/dL with a high reticulocyte count (230,000 cells/mm^3^), hyperleukocytosis with granulocytosis (leukocyte count 16,500 cells/mm^3^, polymorphonuclear cells 9500 cells/mm^3^) and moderate thrombopenia (100,000 cells/mm^3^). Further investigations showed diminished haptoglobin (0.08mg/L), elevated lactate dehydrogenase (4670UI/L) indirect hyperbilirubinemia (21mg/L) with moderate cytolysis and cholestasis (aspartate aminotransferase 43U/L, alanine aminotransferase 65U/L, phenylalanine ammonia lyase 217U/L and γ-glutamyl transpeptidase 188U/L). Hemoglobin (Hb) electrophoresis test results showed Hb S at 50.3 percent, Hb C at 44 percent and Hb A1 at 0 percent, confirming a diagnosis of SCD (hemoglobin S/C).

Our patient’s erythrocyte sediment rate was 110mm/first hour, her C-reactive protein level was 38mg/L (range <6mg/L), fibrinogen was 6.4g/L (24g/L) and serum protein electrophoresis showed a polyclonal IgG 24g/L (range 9 to 13g/L) with normal immunofixation. Results of a chest X-ray were normal. Abdominal ultrasonography, transthoracic and transesophageal echocardiography results were also normal. A thoraco-abdominal scan revealed numerous splenic infarctions. The results of a bone scan showed diffuse bone infarcts.

Her symptoms were attributed to SCD and hence our patient received blood transfusions, antibiotics and analgesics, but with no improvement. Her fever and arthritis failed to respond to this treatment. Instead, the evolution of her condition was marked by the development of arthritis in her hands and relapse of anemia.

Blood culture test results were negative, and the result of a tuberculin skin test was an 8mm induration. There was no BK virus found in repeated sputum and urine examinations, and procalcitonin test results were negative.

Serology test results for human immunodeficiency virus, hepatitis B, hepatitis C, brucellosis and typhoid fever were all negative. Cytobacteriological urine analysis revealed no bacteria but microscopic hematuria (670 cells/mm^3^) and leukocyturia (50 cells/mm^3^). Proteinuria results were negative.

The results of a Coombs test performed on admission were strongly positive for IgG. Immunological investigations revealed a positive anti-nuclear antibody (1/2600) result, and a positive anti-Sm result. Anti-DNA antibody tests were negative. A test for anti-extractable nuclear antigen antibodies (anti-ENA) was negative. C3 levels and C4 levels were normal (respectively, 0.95g/L and 0.3g/L). Tests for anti-phospholipid antibodies were negative. A diagnosis of SLE associated to SCD was established, with five of the diagnostic criteria of the American College of Rheumatology being met. Steroids were administered as a pulse of methylprednisolone 1g/day for three days followed by oral prednisone at 1mg/kg/day with hydroxychloroquine. Her symptoms quickly improved. At her 18-month follow up, she was in clinical remission on prednisone 5mg per day and hydroxychloroquine; she had not experienced a sickle-cell crisis and her lupus is still quiescent.

## Discussion

In the present report we described the case of a Moroccan woman with SCD and coexistent SLE. The overlap of SLE and SCD is of interest, but the limited number of patients that have been reported previously implies that the association is uncommon [3]. Only 40 similar cases have been reported in the literature over the last 50 years [2-16] (Table 1). The African/Afro-Caribbean/African-American population is predisposed to contracting both SCD and SLE, explaining the fact that most patients with this association are African women (70 percent in Table 1 and 73 percent in the series by Michel *et al*.). All reported cases were relatively young at the time of lupus diagnosis (mean age 23 years, range eight to 57 years). All of them had SCD several years before SLE. Articular involvement is the most frequent lupus-related symptom, present in 84 percent of cases, followed by serositis (36 percent), and glomerulonephritis class III or IV (11 percent). Cutaneous manifestations are not frequently mentioned. Positive anti-nuclear antibody (ANA) results were found in 34 cases. Prognosis was favorable in 80 percent of cases (Table 1). Patients with SCD present with a defective activation of the alternate pathway of the complement system; this is the reason why these patients are at increased risk of capsulate bacteria infection, such as from pneumococci [15]. Some authors have suggested the hypothesis that this defect may lead to immune complex disorders secondary to failure to eliminate antigens, predisposing these patients to autoimmune diseases, but this has not been confirmed in other studies [3,11,13]. The clinical features of SLE and SCD have certain elements in common. Diverse manifestations such as polyarthritis, anemia, fever, visceral pain, renal, cardiovascular and pulmonary involvement are common in both conditions. Owing to the overlap of clinical features in the two diseases it may easy to confuse them, as occurred with our patient.

**Table 1:** **Summary of previous case reports of SCD and SLE**[2-16]

**Lead author/year/reference**	**Sex/origin**	**Age of SCD onset**	**Age of SLE onset**	**SLE features**	**Immunologic features**	**Hemoglobin type**	**Treatment**	**Outcome**
Cherner 2010 [3]	F/Afro-Caribbean	13	21	Arthritis, fever	ANA+	SS	Prednisone	Clinical improvement
				Malar rash	Anti-CCP+		Methotrexate	
				Gut vasculitis	Anti-RNP+		Rituximab	
					ACL+		Cyclophosphamide	
Cherner 2010 [3]	F/Afro-Caribbean	7	41	Skin rash	ANA+	SS	Prednisone	Clinical improvement
				Renal disease (biopsy not performed)	Anti-DNA+			
					Anti-Ro+			
Appenzeller 2008 [4]	F/African-American	NA	16	Fever, arthritis	ANA+	SS	Prednisone	Clinical improvement
				Photosensitivity	Anti-SM+		Azathioprime	
				Cardiomyopathy				
				Pericarditis				
Appenzeller 2008 [4]	F/African-American	15	21	Arthritis	ANA+	SS	Prednisone	Clinical improvement
				Pleuritis	Anti-DNA+	SS	Hydroxychloroquine	
				Lymphadenopathy	Anti-Sm+			
Appenzeller 2008 [4]	F/African-American	NA	57	Arthritis	Anti-Sm+	SS	Prednisone	
				Photosensitivity			Hydroxychloroquine	Clinical improvement
				Discoid lesions				
				Raynaud’s phenomenon				
Michel 2008 [2]	F/NA	NA	30	Arthritis	ANA+	SS	Prednisone	Deceased
				Pericarditis	Anti-DNA+			
				Pleuritis	Anti-SSA+			
				GN class II				
Michel 2008 [2]	M/NA	NA	40	Arthritis	ANA+	SS	Prednisone	Remission
				Discoid lesions			Hydroxychloroquine	
				Thrombocytopenia				
Michel 2008 [2]	F/NA	NA	32	Thrombocytopenia	ANA+	SC	Hydroxychloroquine	Remission
					Anti-DNA+			
Michel 2008 [2]	F/NA	NA	35	Arthritis	ANA+	SS	Prednisone	Deceased
				Cutaneous vasculitis	Anti-DNA+		Hydroxychloroquine	
				Raynaud’s phenomenon	Anti-Sm+		Methotrexate	
				GN class II	Anti-SSA+			
					Anti-RNP			
Michel 2008 [2]	F/NA	NA	27	Arthritis	ANA+	SS	Prednisone	Remission
					Anti-DNA+		Hydroxychloroquine	
Michel 2008 [2]	F/NA	NA	25	Arthritis	ANA+	SS	Prednisone	Remission
				GN class III	Anti-DNA+		Hydroxychloroquine	
				Jaccoud arthropathy	ACL+			
				Major depression				
Michel 2008 [2]	M/NA	NA	26	Arthritis	ANA+	SC	Hydroxychloroquine	Clinical improvement
					Anti-DNA+			
					Anti-RNP+			
					ACL+			
Michel 2008 [2]	F/NA	NA	28	Arthritis	ANA+	SS	Prednisone	Persistent renal disease
				GN class IV	Anti-DNA+		Hydroxychloroquine	
				Bullous lupus	Anti-Sm+		Dapsone	
					Anti-RNP+			
Michel 2008 [2]	F/NA	NA	32	Arthritis	ANA+	SS	Prednisone	Remission
				Kikuchi’s disease	RF+			
				Autoimmune hepatitis				
Michel 2008 [2]	F/NA	NA	40	Arthritis	ANA+	SS	Hydroxychloroquine	Clinical improvement
				Discoid lupus	ANA+			
				Venous thrombosis	Anti-Ro+			
					ACL			
Michel 2008 [2]	F/NA	NA	38	Arthritis	ANA+	SS	Prednisone	Clinical improvement
							Hydroxychloroquine	
Michel 2008 [2]	F/NA	NA	17	Arthritis	ANA+	SS	Prednisone	Clinical improvement
				Thrombocytopenia	Anti-Ro+		Hydroxychloroquine	
					Anti-La+			
					ACL+			
Michel 2008 [2]	F/NA	NA	35	Pedal and peri-orbital edema	ANA+	SC	Prednisone	Dialysis
				Ascites and renal failure	Anti-DNA+		Cyclophosphamide	
				GN class IV				
Oqunbiyi 2007 [6]	M/African	NA	8	Malar rash			Prednisone	Clinical improvement
				Arthritis			Hydroxychloroquine	
				Seizures				
				Fever				
Khalide 2005 [7]	F/NA	16	24	Heart failure	Anti-DNA+	SC	Prednisone	Clinical improvement
				Renal failure	Anti-Sm+			
				Pericarditis	Lupus anticoagulant+			
				Pulmonary emboli				
				Polyneuropathy				
				Generalized seizures				
Khalide 2005 [7]	M/NA	NA	16	Discoid rash	ANA+	SS	Prednisone	Clinical improvement
				Polyarthritis	Anti-DNA+		Hydroxychloroquine,	
				Partial seizures			azathioprine	
Khalide 2005 [7]	M/NA	NA	23	Skin rash	ANA+	SS	Hydroxychloroquine	Lost to follow up
				Pleuritis	ACL+			
				Arthritis				
				Raynaud’s phenomenon				
Khalide 2005 [7]	F/NA	NA	28	Arthritis	ANA+	SS	Prednisone	Clinical improvement
				Oral ulcers	Anti-DNA+			
				GN class III	ACL			
Saxena 2003 [8]	M/African-American	NA	9	Arthritis	ANA+	SS	Prednisone	Clinical improvement
				Fever	Anti-DNA+		Cyclophosphamide	
				Acute chest syndrome	Anti-SSA+			
				Pericarditis				
				Seizures				
Saxena 2003 [8]	F/African-American	NA	7	Fever	ANA+	SS	Prednisone	Clinical improvement
				Arthritis	Anti-DNA+		Cyclophosphamide	
				Alopecia			Azathioprine	
				GN class II				
Saxena 2003 [8]	F/African-American	NA	11	Fever	ANA+	SS	Prednisone	Clinical improvement
				Arthritis			Cyclophosphamide	
				Skin rash				
				Seizures				
				Cardiomegaly				
Saxena 2003 [8]	F/African-American	NA	14	Seizures	ANA+	SS	Prednisone	Septic shock due to pneumococcal bacteremia
				Malar rash	Anti-DNA+		Cyclophosphamide	
				Splenomegaly			Azathioprine	
				Arthritis			Plasmapheresis	
				Pericarditis			Splenectomy	
Saxena 2003 [8]	M/African-American	NA	17	Malar rash	ANA+	SS	Prednisone	Hemodialysis dependent
				Alopecia			Cyclophosphamide	
				Pericarditis				
				Cardiomegaly				
				GN class V				
Shetty 1998 [9]	F/Afro-Carribbean	Nine months	10	Arthritis	LE cells in pericardial effusion	SS	Prednisone	Clinical improvement
				Pulmonary infiltrate				
				Pericarditis				
				Myocarditis				
Pham 1997 [10]	F/Afro-Caribbean	NA	18	Arthritis	ANA+	SS	Prednisone	Clinical improvement
				Nephrotic syndrome	Anti-DNA			
Katsanis 1987 [11]	F/Afro-Caribbean	NA	16	Arthritis	ANA+	SS	Prednisone	Clinical improvement
				Malar rash	Anti-DNA+		Hydroxychloroquine	
				Photosensitivity	Anti-Sm+			
				Pleuritis				
				Pericarditis				
				Renal class II				
Katsanis 1987 [11]	F/Afro-Caribbean	NA	15	Arthritis	ANA+	SS	Prednisone	Clinical improvement
				Pleuritis	Anti-DNA borderline	SC	Prednisone	Clinical improvement
Warrier 1984 [12]	F/Afro-Caribbean	NA	11	Malar rash	ANA+			
				Alopecia	Anti-DNA+			
				Arthralgia	Anti-ENA+			
				Seizures				
				Hepatosplenomegaly				
Luban 1980 [13]	F/African-American	NA	8	Discoid lesions	Positive LE	SC	Prednisone	Clinical improvement
				Pericarditis	ANA+			
				Myocarditis				
Luban 1980 [13]	F/African-American	NA	14	Fever	ANA+	SS	Prednisone	Clinical improvement
				Renal disease	Positive LE			
Karthikeyan 1978 [14]	F/African	4	15	Arthritis	ANA+	SS	Prednisone	Clinical improvement
				Raynaud’s phenomenon	Positive LE cell test			
				Photosensitivity				
Wilson 1976 [15]	F/African-American	30	40	Arthritis	Positive LE cells	SS	Prednisone	Deceased
				Pleuritis				
				Libman-Sacks endocarditis				
Wilson 1976 [15]	F/African-American	Four months	16	Arthritis	ANA+	SS	Prednisone	Clinical improvement
				Hepatitis	Anti DNA +			
				Pneumonitis				
Wilson 1976 [16]	F/African-American	NA	27	Arthritis	Histopathologic evidence for SLE on post-mortem examination	SS	No treatment for SLE	Deceased
				Malar rash				
				Pulmonary congestion				
				Hepatomegaly				
				Nephrotic syndrome				
				Cerebral and subarachnoid hemorrhage				

ACL=anti-cardiolipin antibodies; ANA=anti-nuclear antibodies; anti-ENA=anti-extractable nuclear antigen antibodies; GN=glomerulonephritis; NA=not available; RF=rheumatoid factor; anti-RNP=anti-ribonucleoprotein antibodies; SCD=sickle-cell disease; SLE=systemic lupus erythematosus; anti-SSA=anti-Sjögren syndrome antigen A antibodies.

Further, the frequency and titers of antibodies in SCD have been reported as relatively higher than in population controls, making the diagnosis more challenging in clinical practice [17].

Toly-Ndour *et al*. reported that 50 percent of 88 patients with SCD had positive anti-nuclear antibody results and 20 percent had titers greater than one in 200, but only one patient developed rheumatoid arthritis five years later and no patients developed SLE [18]. In this series, patients treated with hydroxyurea had ANA-positive results less frequently than non-treated patients (P=0.053) [18].

Large prospective epidemiological studies are necessary to determine whether the prevalence of immune complex diseases is increased in patients with SCD.

## Conclusions

This report illustrates the importance of considering associated diseases when clinical findings are unexplained by SCD alone, or are unresponsive to the conventional treatment. Early diagnosis and the initiation of appropriate treatment may decrease morbidity and mortality in these patients.

## Consent

Written informed consent was obtained from the patient for publication of this case report and any accompanying images. A copy of the written consent is available for review by the Editor-in-Chief of this journal.

## Competing interests

The authors declare that they have no competing interests.

## Authors’ contributions

MM was the major contributor to the writing of the manuscript. ZTM reviewed the manuscript and prepared the final draft. HH and WM made substantial contributions to the acquisition and interpretation of clinical data and performed the literature research in PubMed. MAd and MAo gave final approval for the version to be published. All authors read and approved the final manuscript.
